# Temporal effects of maternal psychological distress on child mental health problems at ages 3, 5, 7 and 11: analysis from the UK Millennium Cohort Study

**DOI:** 10.1017/S0033291718001368

**Published:** 2018-06-11

**Authors:** Steven Hope, Anna Pearce, Catherine Chittleborough, Jessica Deighton, Amelia Maika, Nadia Micali, Murthy Mittinty, Catherine Law, John Lynch

**Affiliations:** 1 UCL Great Ormond Street Institute of Child Health, London, UK; 2 School of Public Health, University of Adelaide, Adelaide, Australia; 3 UCL and Anna Freud National Centre for Children and Families, London, UK; 4 Faculty of Social and Political Science, Gadjah Mada University, Yogyakarta, Indonesia; 5 School of Social and Community Medicine, University of Bristol, Bristol, UK

**Keywords:** Child development, epidemiology, life course, maternal factors

## Abstract

**Background:**

Psychological distress is common among women of childbearing age, and limited longitudinal research suggests prolonged exposure to maternal distress is linked to child mental health problems. Estimating effects of maternal distress over time is difficult due to potential influences of child mental health problems on maternal distress and time-varying confounding by family circumstances.

**Methods:**

We analysed the UK Millennium Cohort Study, a nationally representative sample with data collected throughout childhood. Adopting a marginal structural modelling framework, we investigated effects of exposure to medium/high levels of maternal psychological distress (Kessler-6 score 8+) on child mental health problems (Strengths and Difficulties Questionnaire borderline/abnormal behaviour cut-off) using maternal and child mental health data at 3, 5, 7 and 11 years, accounting for the influence of child mental health on subsequent maternal distress, and baseline and time-varying confounding.

**Results:**

Prior and concurrent exposures to maternal distress were associated with higher levels of child mental health problems at ages 3, 5, 7 and 11 years. For example, elevated risks of child mental health problems at 11 years were associated with exposure to maternal distress from 3 years [risk ratio (RR) 1.27 (95% confidence interval (CI) 1.08–1.49)] to 11 years [RR 2.15 (95% CI 1.89–2.45)]. Prolonged exposure to maternal distress at ages 3, 5, 7 and 11 resulted in an almost fivefold increased risk of child mental health problems.

**Conclusions:**

Prior, concurrent and, particularly, prolonged exposure to maternal distress raises risks for child mental health problems. Greater support for mothers experiencing distress is likely to benefit the mental health of their children.

## Introduction

Childhood mental health problems are common, with consequences that may extend into adulthood. In the UK, the British Child and Adolescent Mental Health Surveys carried out in 1999 and 2004 (the most recent national data available) found that prevalence of diagnosable mental disorder was 10% for children between the ages of 5 and 16 (Green *et al.*, 2005). Apart from individual and family distress, and current public health burden, mental health problems in childhood can have sequelae later in life, with increased risks for mental health problems and poorer outcomes in physical health, education, employment and social domains (Murphy and Fonagy, 2013). The substantial direct and indirect costs to society of childhood mental illness have led to a focus on early prevention (Department of Health, 2015).

Among women of childbearing age, symptoms of distress are also common (Goodman, 2007). Population prevalence of common mental disorder symptoms ranges from a fifth to a quarter in England for women between ages 16 and 44 years (Stansfeld *et al.*, 2016). A large body of research has shown that maternal distress is a predictor of child and adolescent mental health problems (Goodman *et al.*, 2011), with risks extending to adult mental health (Weissman *et al.*, 2016). However, much of the published research has been based on small or clinical samples, and has used cross-sectional data or has only had a short follow-up period. Few studies have considered the effects of duration of exposure to maternal distress on child mental health, although distress is frequently chronic or recurrent (Burcusa and Iacono, 2007). From studies that have investigated duration of exposure, there is evidence that, compared with isolated episodes of maternal distress, repeated or prolonged exposure predicts poorer psychological outcomes in children (Fihrer *et al.*, 2009; Turney, 2011*a*) and adolescents (Hay *et al.*, 2008; Murray *et al.*, 2011; Korhonen *et al.*, 2014; Mikkonen *et al.*, 2016). Most evidence for an association between maternal distress and child mental health has involved diagnosed severe or treated maternal distress. There is a paucity of research on the effects of moderate distress, although this limited evidence base suggests that even lower levels of maternal distress may predict poor child mental health (Prady *et al.*, 2016), particularly if exposure has been prolonged (Hammen and Brennan, 2003; Cents *et al.*, 2013).

Existing research on duration of exposure to maternal distress on child mental health problems has not taken into account either time-varying confounding or potential bi-directional associations between child mental health problems and maternal distress. Pathways linking prolonged maternal distress with child mental health are likely to be complex and a number of potential biological, psychosocial and socioeconomic mechanisms for the transmission of maternal distress to child outcomes have been proposed (Elgar *et al.*, 2004). First, there may be reciprocity between the mental health of mother and child, so that in addition to a mother's psychological distress influencing her child's mental health, the child's mental health problems may also have a negative impact on a mother's levels of distress. Although evidence of bi-directional effects is mixed (Elgar *et al.*, 2003; Kouros and Garber, 2010; Raposa *et al.*, 2011; Panico *et al.*, 2014), the influence of child mental health problems on subsequent maternal distress ought to be considered as a mechanism influencing long-term associations between the mental health of the mother and child. Second, wider social determinants may confound relationships between maternal and child mental health. The mental health problems of both mothers and children are influenced by a range of shared risk factors (Moilanen *et al.*, 2010), including low income, family breakdown, non-employment, less secure housing tenure, ethnic minority status and the presence of multiple children in the family (Green *et al.*, 2005; McLaughlin *et al.*, 2011; Reiss, 2013; Roberts *et al.*, 2016; Wickham *et al.*, 2017). In addition, these shared risk factors (referred to subsequently in this paper as ‘family circumstances’) may also be influenced by prior mental health (Kessler *et al.*, 1995).

**Fig. 1.**
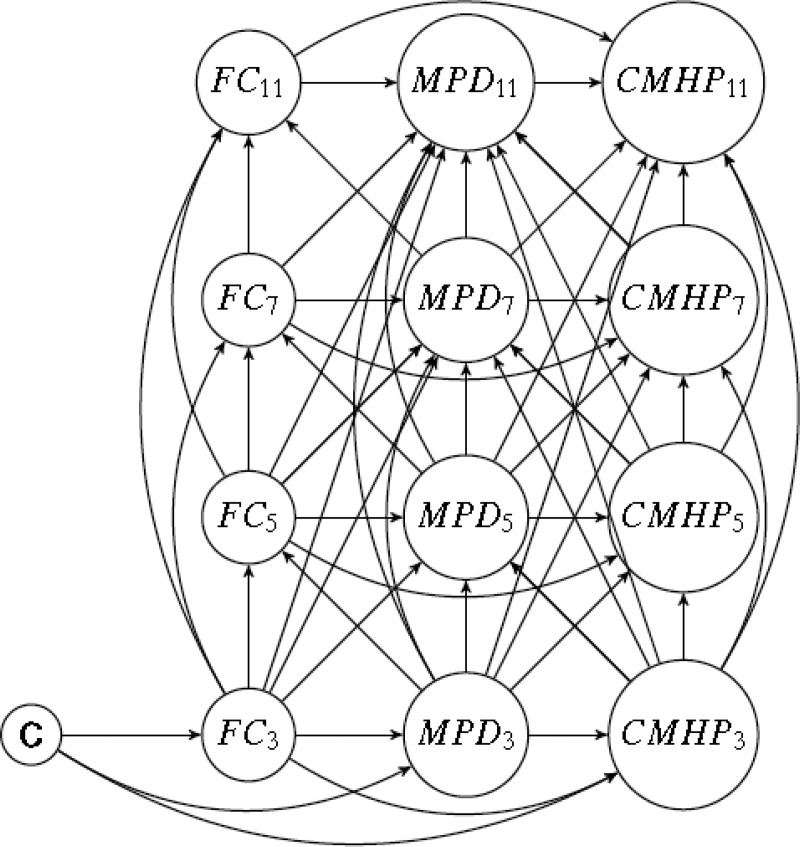
Directed Acyclic Graph (DAG) of hypothesised pathways between maternal psychological distress (K6) and child mental health problems (SDQ) at ages 3, 5, 7 and 11 years. C: baseline confounders (lone parent family, number of children, age at birth of cohort child, maternal highest academic qualification and ethnicity). FC: Family circumstances (latent classes summarising: household poverty, lone parent family, a new sibling in household and maternal employment status). MPD: Maternal psychological distress (K6). CMHP: Child mental health problems (SDQ).

Recent advances in causal modelling have shown that standard statistical methods to adjust for time-varying confounding that is influenced by previous exposure can lead to biased estimates for two reasons (VanderWeele, 2015). First, conditioning on a confounder that is affected by previous exposure removes part of the effect of interest (Daniel *et al.*, 2011). For example, in estimating the effect of maternal distress on child mental health adjustment for family circumstances (a time-varying confounder) using standard regression accounts for its confounding influence as a common cause of maternal distress (the exposure) and child mental health problems (the outcome). However, maternal distress is also a cause of later family circumstances. In other words, part of the effect of maternal distress works through later family circumstances. Conditioning in standard regression blocks the confounding pathway from maternal distress to child mental health that acts via family circumstances, but in doing so underestimates the effect of maternal distress on child mental health. Second, if there are unmeasured factors associated with both family circumstances and child mental health, conditioning on a confounding variable (as is done in standard regression) induces further confounding through unmeasured pathways from exposure to outcome via these unmeasured variables. This is known as ‘collider stratification bias’ (Hernan *et al.*, 2004). Consistent with recent innovations in methods for complex time-varying data structures (Daniel *et al.*, 2011), we used a marginal structural model (MSM) (Robins *et al.*, 2000) to account for the complex hypothesised pathways between exposure (maternal distress), outcome (child mental health), baseline confounding and time-varying confounding by family circumstances, as shown in Fig. 1, using observational data from a nationally representative cohort study.

## Methods

### Subjects and design

We used data from the Millennium Cohort Study (MCS), a longitudinal study of children born in the UK between September 2000 and January 2002, which has been described elsewhere (Connelly and Platt, 2014). Ethical approval for the MCS was received from a Research Ethics Committee at each sweep (Hansen, 2014). Data were obtained from the UK Data Archive, the University of Essex, in March 2014.

The first study contact with the cohort child was at 9 months, with survey interviews carried out by trained interviewers in the home with the main respondent (usually the mother) and their partner, where present. Information was collected on 72% of those approached, providing information on 18 818 infants (our analyses were restricted to 18 296 singletons). At the time of carrying out these analyses, data had been collected at a further four sweeps, when the children were aged 3, 5, 7 and 11 years. By 11 years the number of families who had participated in all five sweeps had declined to 10 313 (56% of the respondents who had taken part at the 9 months sweep). Of these families, only 4610 had complete data on all exposure, outcome and baseline or time-varying confounder variables from ages 3 to 11 years.

Multiple imputation was carried out on missing exposure, outcome and confounder data for the 18 296 singleton children who were included in the original 9-month sweep of data collection. As multiple observations of exposure and outcomes would be expected to correlate, we imputed scales (Kessler-6 and Strengths and Difficulties, respectively), setting a minimum threshold of having scores in at least two of the four sweeps. This reduced the analytic sample to 12 201 families, with missing data imputed for 7591 of these families, using the Stata/SE 13 (Stata Corporation, TX) ‘mi impute chained’ command to create 10 imputed datasets.

Table 1 shows frequency distributions for variables included in the analyses if any data were recorded (observed sample, no restrictions), the multiply imputed sample, and also a complete case sample. Compared with the observed sample, prevalences of markers of disadvantage and maternal or child mental health problems were generally somewhat lower in the multiply imputed sample (used for analyses reported in this paper) and considerably lower in the complete case sample.

**Table 1.** Frequency distributions of study variables, according to sample

	Observed sample (no restrictions) (*n* = 18 296)	Multiply imputed sample (*n* = 12 201)	Complete case sample (*n* = 4610)
*Sex of child*			
Male	51.5 (9415)	50.5	49.1 (2263)
Female	48.5 (8881)	49.5	50.9 (2347)
*Baseline confounders (9 months)*			
Maternal highest qualification			
Degree	15.7 (2860)	19.3	25.0 (1153)
Diploma	8.4 (1525)	9.7	11.3 (519)
A/AS/S levels	9.3 (1696)	10.6	12.4 (572)
O levels/GCSEs A-C	33.5 (6100)	34.9	36.4 (1584)
O levels/GCSEs D-G	10.8 (1961)	10.1	8.2 (376)
Other/none	22.4 (4087)	15.4	8.8 (406)
*Item missing*	*67*		
Number of children			
1 child	42.1 (7695)	42.7	42.2 (1943)
2 children	34.5 (6320)	35.5	38.6 (1777)
3 children	20.7 (3795)	19.9	18.2 (839)
4+ children	2.7 (486)	1.9	1.1 (51)
Maternal age at cohort child's birth			
14–19 years	8.7 (1589)	7.5	5.1 (234)
20–29 years	46.9 (8579)	44.2	40.2 (1855)
30–39 years	42.3 (7736)	46.0	52.1 (2400)
40 years and above	2.1 (389)	2.3	2.6 (121)
*Item missing*	*3*		
Ethnicity			
White	83.9 (15 315)	90.4	95.7 (4411)
Indian	2.6 (476)	1.9	0.9 (43)
Pakistani/Bangladeshi	6.9 (1256)	3.3	1.2 (53)
Black/Black British	3.7 (667)	2.5	1.2 (57)
Other/mixed	2.9 (533)	1.8	1.0 (46)
*Item missing*	*49*		
Lone parent family			
Two parents	82.7 (15 128)	85.4	90.1 (4153)
One parent	17.3 (3160)	14.6	9.9 (457)
*Item missing*	*8*		
*Time-varying confounders*			
Family circumstances at 3 years			
Adverse circumstances	32.4% (4761)	29.1%	19.4% (894)
Not Adverse	67.6% (9935)	70.9%	80.6% (3716)
*Not present at relevant sweep* (*3 years*)	*3600*		
Family circumstances at 5 years			
Adverse circumstances	33.4% (4828)	28.7%	19.1% (881)
Not Adverse	66.7% (9650)	71.3%	80.9% (3729)
*Not present at relevant sweep* (*5 years*)	*3818*		
Family circumstances at 7 years			
Adverse circumstances	29.5% (3891)	26.0%	17.7% (814)
Not Adverse	70.5% (9301)	74.0%	82.3% (3796)
*Not present at relevant sweep* (*7 years*)	*5104*		
Family circumstances at 11 years			
Adverse circumstances	22.6% (2858)	19.1%	11.5% (529)
Not Adverse	77.4% (9784)	80.9%	88.5% (4081)
*Not present at relevant sweep* (*11 years*)	*5654*		
*Exposure variables*			
K6 at 3 years			
No	88.5 (11 580)	89.2	91.8 (4230)
Yes	11.5 (1503)	10.8	8.2 (380)
*Scale items missing*	*1613*		
*Not present at relevant sweep* (*3 years*)	*3600*		
K6 at 5 years			
No	88.2 (12 032)	89.2	91.8 (4232)
Yes	11.8 (1617)	10.8	8.2 (378)
*Scale items missing*	*829*		
*Not present at relevant sweep* (*5 years*)	*3818*		
K6 at 7 years			
No	88.4 (10 781)	89.2	92.1 (4246)
Yes	11.6 (1413)	10.8	7.9 (364)
*Scale items missing*	*998*		
*Not present at relevant sweep* (*7 years*)	*5104*		
K6 at 11 years			
No	82.7 (9187)	83.6	87.9 (4054)
Yes	17.3 (1923)	16.4	12.1 (556)
*Scale items missing*	*1532*		
*Not present at relevant sweep* (*11 years*)	*5654*		
*Outcome variables*			
SDQ at 3 years			
No	82.5 (7926)	82.2	87.2 (4018)
Yes	17.5 (1676)	17.8	12.8 (592)
*Scale items missing*	*5094*		
*Not present at relevant sweep* (*3 years*)	*3600*		
SDQ at 5 years			
No	90.4 (10 044)	90.4	94.0 (4332)
Yes	9.6 (1065)	9.6	6.0 (278)
*Scale items missing*	*3369*		
*Not present at relevant sweep* (*5 years*)	*3818*		
SDQ at 7 years			
No	88.3 (9332)	88.1	92.3 (4255)
Yes	11.7 (1242)	11.9	7.7 (355)
*Scale items missing*	*2618*		
*Not present at relevant sweep* (*7 years*)	*5104*		
SDQ at 11 years			
No	86.2 (9551)	86.6	90.4 (4166)
Yes	13.8 (1534)	13.4	9.6 (444)
*Scale items missing*	*1557*		
*Not present at relevant sweep* (*11 years*)	*5654*		

Those families where the mother reported both distress and child mental health problems at 3 years were less likely to participate in all subsequent sweeps compared with those with neither distress nor child mental health problems, although a majority of both groups of families participated in all sweeps (63% *v.* 74%, respectively). This pattern of differential participation in all MCS sweeps was also shown for distress/child mental health problems reported cross-sectionally at 5 years through to 11 years. At all ages, cross-sectional risks of child mental health problems associated with maternal distress were of similar magnitudes in samples with no restrictions for missing data (the observed sample) compared with the multiply imputed sample, with slightly higher risks in the complete case sample. For example, at 3 years, risks of child mental health problems were: observed data: RR 2.94 (2.61–3.31); multiply imputed sample: 2.79 (2.53–3.07); complete case sample: 3.42 (2.82–4.13). At 11 years, risks of child mental health problems were: observed sample: RR 3.47 (3.12–3.86); multiply imputed sample: 3.38 (3.08–3.72); complete case sample: 3.87 (3.18–4.71). Results from MSM analyses carried out using the complete case sample (not shown) were similar to those reported in the paper using the multiply imputed sample.

Stata/SE 13 was used for all analyses. ‘Svy’ commands were employed to account for the sampling design of the MCS when calculating weighted percentages and means.

### Measures

Measurement of child mental health problems and maternal psychological distress in the MCS using consistent, validated measures started when the child was aged 3 years, with follow-up measurement using the same scales at each subsequent sweep.

#### Child mental health problems

Mental health problems were assessed at 3, 5, 7 and 11 years using the Strengths and Difficulties Questionnaire (SDQ) (Goodman, 1997), a 25-item measure completed by the mother. We used the total difficulties score, the sum of four difficulties scales (peer problems, conduct disorders, hyperactivity and emotional problems) to classify children, using validated cut-offs, for ‘normal’ (0–13) or ‘borderline-abnormal’ scores (14–40). As sensitivity analyses, we repeated analyses using the continuous SDQ total difficulties scores and separate subscale scores for Internalising (emotional problems and peer problems) and Externalising (hyperactivity and conduct disorders) behaviour. Patterns of results for analyses of continuous SDQ scores and subscales replicated those reported here for the dichotomous total difficulties score.

#### Maternal psychological distress

Psychological distress was measured at 3, 5, 7 and 11 years using the Kessler-6 scale (K6) (Kessler *et al.*, 2003), a six-item measure completed by the mother, asking how often during the past 30 days they felt: ‘so depressed that nothing could cheer you up’, ‘hopeless’, ‘restless or fidgety’, ‘that everything was an effort’, ‘worthless’ and ‘nervous’. Each item had a five-point response, from ‘None of the time’ (0) to ‘All of the time’ (4). Responses to each item were combined to produce a single score ranging from 0 to 24. Scores at each sweep were dichotomised using an established cut-off for medium/high maternal distress (a score or eight or more on the K6) (Center for Disease Control, 2016). The cut-off was chosen to identify common, moderate distress as well as more serious mental illness.

#### Baseline confounding

A number of potential confounders were identified, all recorded at first contact when the child was aged 9 months. These included both family characteristics (lone parent family *v.* other; the number of children in the household) and maternal characteristics (highest academic qualification, age at birth of cohort child and ethnicity).

#### Time-varying confounding

At sweeps from 3 through to 11 years the following potential time-varying confounders were identified: household poverty (net equivalised family income, before housing costs, below 60% of the national median), lone parent family (lone parent *v.* two-parent family), new child or children born to the mother between consecutive sweeps, and maternal employment (non-employment *v.* other). Within an MSM, because weighting is used to standardise the exposure, it is only technically possible to include a single time-varying confounding (weighting) variable at each sweep. Thus, information from all potential confounding variables has to be summarised into a single variable that can then be used in the weighting (or standardisation) procedure. Therefore, these confounding variables were combined to derive family circumstances variables, using Latent Class Analysis (LCA) to summarise cross-sectional relations between the variables at ages 3, 5, 7 and 11 years. Two and three-class solutions were assessed for model fit and interpretability (online Supplementary Appendix S1). A two-class solution fitted the data well at all sweeps, and reflected a higher probability of adverse family circumstances (*v.* not experiencing these circumstances). LCA was carried out using a Stata plug-in for the SAS procedure PROC LCA.

### Analyses

Descriptive analyses were carried out to examine relationships between maternal psychological distress and child mental health problems. We plotted the proportion of children with a mental health problem according to a number of sweeps at which the mother had reported medium or high distress.

### Modelling the effects of exposure to maternal psychological distress on child mental health problems, accounting for time-varying confounding

We estimated the effects of exposure to maternal psychological distress on child mental health problems, taking into account time-varying confounding. To properly account for time-varying relationships between confounders, exposures and outcomes (illustrated in Fig. 1) we used the inverse probability of treatment weighting (IPTW) of an MSM (Robins *et al.*, 2000). Technical details about the MSM are shown in online Supplementary Appendix S2. Separate weights were calculated for each sweep from 3 to 11 years, including the influence of prior child mental health and confounding pathways identified for that sweep. The weights produced for each sweep were then combined in a single weight (95% truncated to reduce the influence of outliers). No statistically significant interactions were observed between child's sex and maternal distress in the models, and therefore analyses are for boys and girls combined.

We adopted an analytic approach reported by VanderWeele *et al.* (2011). We carried out separate MSM analyses for the effects of prior and concurrent exposure to maternal psychological distress on child mental health problems (the outcome) at ages 3, 5, 7 and 11 years. Risk ratios (RR) were estimated using generalised linear models, and robust variance estimation was used for standard errors to account for sampling error in the estimation of the weights. To illustrate, for child mental health problems at 3 years we estimated the effect of maternal distress at 3 years, accounting for confounders at baseline (9 months) and 3 years only. For child mental health problems at 5 years, we estimated the joint effects of maternal distress at 3 and 5 years, accounting for confounders at baseline, 3 and 5 years, together with the influence of child mental health problems at 3 years on maternal distress at 5 years. The same approach was applied for child mental health problems as an outcome at 7 and 11 years. By 11 years, the MSM estimated the effect of maternal distress at 3, 5, 7 and 11 on child mental health at 11 years, accounting for time-varying confounding from 3 to 11 years. For each age of outcome, effects associated with maternal distress at each age of exposure were summed to provide an estimate of the effect of prolonged exposure to maternal distress. This joint effect of exposures to maternal distress on child mental health problems consists of the combined direct effects of each exposure on the outcome, unmediated by any other exposure: (1) the direct effect of *X*_0_ on *Y* unmediated by {*X*_1_, *X*_2_, …*X*_*t*_} on *Y*; (2) the direct effect of *X*_1_ on *Y* unmediated by {*X*_2_, *X*_3_, …, *X*_*t*_} and so on. For example, the effect on child mental health problems at age 7 years of exposures to maternal distress at ages 3, 5 and 7 are *β*_1_,  *β*_2_ and *β*_3_, and the joint effect is the simple sum of these direct effects (*β*_1_ + *β*_2_ + *β*_3_). Since the outcome variables are log transformed, the joint effect is the exponentiated sum of these effects (‘Summed Risk Ratios’).

### Sensitivity analysis

We carried out an unmeasured confounding sensitivity analysis using methods outlined by Ding and VanderWeele (2016) in order to estimate the magnitude of confounding that would be necessary to explain the observed effects of exposure variables on outcomes. For each observed effect, two relative risks were estimated: between the exposure and the unmeasured confounder and between the unmeasured confounder and the outcome. These exchangeable estimates demonstrate the size of unmeasured confounding required for a relative risk of unity between exposure and outcome. The estimation of large risks from the sensitivity analyses would suggest that the observed exposure–outcome relationship was less likely to be the result of unmeasured confounding (Technical details are shown in online Supplementary Appendix S3).

## Results

The proportion of children reporting mental health problems (an SDQ total difficulties score within the borderline/abnormal range) varied between data collection sweeps: 3 years (20%), 5 years (10%), 7 years (14%) and 11 years (15%). The proportion of mothers identified with psychological distress (a K6 score of eight or more, within the medium or high score range) also varied: 3 years (11%), 5 years (12%), 7 years (12%) and 11 years (17%).

### Accounting for time-varying confounding

The effects of maternal distress on child mental health problems at 3, 5, 7 and 11 years are shown in Table 2, using MSM to properly account for confounding (including prior child mental health problems, maternal distress, time-varying family circumstances and baseline confounders). Risks of child mental health problems were elevated if there had been any prior or concurrent exposure to maternal distress. For example, at 11 years risks of child mental health problems were raised if maternal distress had been reported at any of the data collection sweeps: 3 years [RR 1.27 (1.08–1.49)], 5 years [RR 1.19 (1.01–1.41)], 7 years [RR 1.39 (1.20–1.62)], or 11 years [RR 2.15 (1.89–2.45)]. Although exposure to concurrent maternal distress had the largest effect on child mental health [concurrent exposure: 5 years (RR 2.53 (2.16–2.96)), 7 years (RR 2.30 (1.97–2.67)) or 11 years (RR 2.15 (1.89–2.45))], raised risks were also associated with prior maternal distress, indicating that both recent maternal distress and a history of distress had effects on child mental health problems. Prolonged exposure to maternal distress at all ages (3, 5, 7 and 11 years) was estimated by summing risks of exposure to prior and concurrent maternal distress for each age of outcome and showed an almost fivefold increased risk of child mental health problems at ages 5 (RR 4.88), 7 (RR 4.87) and 11 years (RR 4.52).

**Table 2.** Risks ratios (95% CI) for child mental health problems (SDQ)^a^ by maternal psychological distress (K6)^b^ at ages 3, 5, 7 and 11 years^c^ (*n* = 12 201)

	Risk ratios (95% CI)	Summed risk ratios
Child mental health problems at 3 years		
Maternal distress at 3 years	2.54 (2.30–2.81)	
Child mental health problems at 5 years		
Maternal distress at 3 years	1.93 (1.64–2.28)	4.88
Maternal distress at 5 years	2.53 (2.16–2.96)	
Child mental health problems at 7 years		
Maternal distress at 3 years	1.46 (1.21–1.76)	4.87
Maternal distress at 5 years	1.45 (1.23–1.71)	
Maternal distress at 7 years	2.30 (1.97–2.67)	
Child mental health problems at 11 years		
Maternal distress at 3 years	1.27 (1.08–1.49)	4.52
Maternal distress at 5 years	1. 19 (1.01–1.41)	
Maternal distress at 7 years	1.39 (1.20–1.62)	
Maternal distress at 11 years	2.15 (1.89–2.45)	

a SDQ score within the borderline abnormal range (a score of 14 or more).

b K6 score within medium/high distress range (a score of 8 or more).

c Models account for effects of child mental health problems (SDQ) on subsequent maternal distress (K6); baseline confounders (9 months: lone parent family, number of children, maternal highest academic qualification, ethnicity, maternal age at birth of cohort child) and time-varying confounders (family circumstances).

Results from sensitivity analyses, shown in online Supplementary Table S3.1 (Appendix S3) indicate that substantial unmeasured confounding would be necessary in order to account for all observed exposure–outcome associations reported in Table 2. For example, consider the association between maternal distress at 3 years on child mental health problems at 11 years. Even if there was an unmeasured confounder that had a weak association with maternal distress at 3 years (RR 1.3), this confounder would need to be strongly associated with child mental health problems at 11 years (RR 10) for there to truly be no association (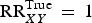) rather than the observed association (
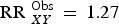
). Although it may be possible to identify an unmeasured confounder associated with *X* (or *Y*) at RR 1.3, it is difficult to conceive of an unmeasured confounder that would have such a strong association with child mental health.

## Discussion

Using longitudinal data from a representative cohort of UK children, we investigated whether exposure to maternal psychological distress at ages 3, 5, 7 and 11 years was associated with child mental health problems assessed over the same period. Analyses were carried out using MSM that could incorporate the influence of a child's mental health problems on subsequent maternal psychological distress and time-varying sociodemographic characteristics that may confound the effects of maternal distress on child mental health. After accounting for time-varying confounding, we found that concurrent, prior and, in particular, prolonged exposure to maternal distress was associated with an increased risk of child mental health problems. Early exposure to maternal distress was found to have an effect on child mental health problems up to eight years later. Although it is not possible to investigate the reason for this result in our study, explanations might include the latent impact of early life programming or vulnerability which is manifest at later ages (Lewis *et al.*, 2014; Vaiserman, 2015).

While other studies have reported associations between maternal psychological distress and offspring mental health (Goodman *et al.*, 2011) we have extended the evidence base in a number of ways. This is the only UK-representative analysis to have investigated the effect of prolonged exposure to maternal distress on child mental health problems from early to mid-childhood that properly accounts for the complex data structure of repeated exposures, outcomes and time-varying confounding. We acknowledge that the impact on the child may vary according to the type and severity of the maternal mental health problems manifested. However, the strength of a population cohort is the ability to investigate mental health at a non-clinical level, and we have focused on maternal psychological distress, which is common in women of childbearing age (Stansfeld *et al.*, 2016). This level of distress, which is socially patterned, might be tackled by means other than clinical interventions, such as through economic and social support for the mother and her family.

We considered whether duration might be a marker of severity of distress, and found this not to be the case. Scores among mothers who reported prolonged distress were not exclusively within the severe range. For example, of the mothers who were distressed at every sweep from 3 to 11 years, the proportion with severe distress at any sweep ranged from 53% at 3 years to 60% at 11 years. Thus, a substantial proportion of distress at all four sweeps was experienced at lower levels of distress.

We employed an innovative methodological approach to attempt to correctly estimate the effects of maternal distress on child mental health problems with other time-varying confounding taken into account. Previous research has shown that socioeconomic and maternal psychological distress are separate risk pathways to child mental health problems (Mensah and Kiernan, 2010; Woolhouse *et al.*, 2015; Mikkonen *et al.*, 2016), while the evidence with regard to whether child mental health problems predict subsequent maternal psychological distress is conflicting (Elgar *et al.*, 2003; Garber and Cole, 2010; Kouros and Garber, 2010; Garber *et al.*, 2011; Raposa *et al.*, 2011; Panico *et al.*, 2014). Our findings suggest that the effects of maternal psychological distress are strong after accounting for time-varying sociodemographic factors (confounding) and the influence of prior child mental health problems on maternal distress.

### Strengths and limitations

Our analyses were based on an *a priori* model of the hypothesised relationship between exposure to maternal distress and child mental health problems. The model explicitly identified associations between variables of interest, which could be directly represented in our analytic framework. We used MSMs to estimate the effects of maternal psychological distress on child mental health problems accounting for time-varying confounding, including complex temporal relationships between variables. MSMs allow us to model causal relations as a specification of pathways between variables of interest in the hypothetical model that is estimated. However, MSMs are not the only method which can be used to estimate causal models, and it is noted that recent research provides guidance on how SEMs might incorporate a potential outcomes framework within a mediation analysis at a single time point (Muthén and Asparouhov, 2015; Coman et al., 2017). Nevertheless, our use of MSM analyses, explicitly incorporating temporal relationships between confounding, exposure and outcome variables (such as exposure-induced intermediate confounding), reduced the possibility of biased estimates (Daniel *et al.*, 2011). Importantly, this approach allowed us to use all available data on exposure, outcome and confounding from ages 3–11 to estimate prior, concurrent and prolonged effects of maternal distress on child mental health. We used the UK MCS, a large, nationally representative contemporary cohort of UK children with data on maternal psychological distress, child mental health problems and a wide range of sociodemographic factors measured from early childhood to age 11 years. The breadth of information recorded in the MCS over such a length of time (including repeat measurement of K6 and SDQ over four sweeps) differentiates this dataset from many other studies used to investigate the relationship between maternal psychological distress and child mental health problems.

Using observational data to aid causal interpretation is only possible under very strict assumptions. These include that there is no unmeasured exposure–outcome confounding, no unmeasured mediator–outcome confounding, no unmeasured exposure–mediator confounding, and no mediator–outcome confounder affected by exposure (Valeri and Vanderweele, 2013), where time-varying confounders may also take on the role of mediator as specified in the model tested (see Fig. 1). The adoption of a causal modelling approach cannot establish that a theoretical model represents true causal relations. However, if the theoretical model has been correctly specified it will aid a causal interpretation. In this case, we developed an *a priori* model, derived from theory and research, in order to investigate relationships between maternal distress and child mental health problems over time, accounting for confounding.

Attrition and missing data are problems common to all longitudinal studies. 56% of MCS singletons participated in all the sweeps up to 11 years and there was missing data for model variables at each sweep. Therefore, to address bias resulting from non-response we carried out analyses with multiply imputed data (Sterne *et al.*, 2009). We were also constrained by available data. Although MCS recorded information on a wide range of variables relevant to our hypothesised model, it is acknowledged that there may be other potentially influential factors which were either not recorded or which could have been assessed more comprehensively. First, most data were a maternal report, including ratings of her own levels of distress and her child's behaviour at each sweep. Ratings given by mothers (including distressed mothers) can be reliable measures of child mental health problems. Nevertheless, shared method variance is a potential problem when there is a single reporter, particularly as distressed mothers are more likely than non-distressed mothers to view their child's behaviours negatively (Najman *et al.*, 2001). As a consequence, associations between maternal distress and child mental health problems may have been inflated in this study, and, given the multiple sweeps involved in this analysis, this effect may have accumulated over time. However, the sensitivity analysis of residual confounding shows that the bias effects of shared method variance would have to be large to explain away the effects estimated here. The K6 and SDQ are both validated measures, and we carried out sensitivity analyses of SDQ scales (separately for internalising and externalising behaviour, and with continuous scores rather than cut-offs for borderline or abnormal scores), which largely replicated the main findings reported. Second, the initial data collection sweep was carried out when the child was aged 9 months, and the standardised repeated measurement of maternal psychological distress and child mental health problems were only recorded from the 3 years sweep. Therefore, it was not possible to test the influence of an early developmental period using the MCS. Thirdly, our focus on maternal distress does not diminish the possible influence of paternal factors on child mental health problems. Paternal distress is related to child mental health (Connell and Goodman, 2002; Elgar *et al.*, 2007), and when both parents are distressed risk for child mental health problems may be particularly elevated (Mikkonen *et al.*, 2016). Conversely, fathers may be a significant source of support for mothers experiencing distress, and thereby ameliorate negative effects for children (Mezulis *et al.*, 2004; Goodman, 2007). However, a focus on mother–child mental health is justifiable. Mothers are likely to be the main carers for children over the period of the study (Bianchi and Milkie, 2010; Craig and Mullan, 2011), and data on fathers is far less complete than for mothers in the MCS. Finally, we were unable to investigate shared maternal–offspring genetic factors. Evidence from a range of study designs, including family, twin and adoption studies suggests weak direct genetic influences on child mental health (Rice, 2009), with GWAS (genome-wide association studies) into the influence of genes on complex behavioural outcomes showing relatively small effects (odds ratios in the region of 1.1) (Dick, 2011). Results from sensitivity analyses indicated that the degree of unmeasured confounding required to explain the observed exposure–outcome associations was large, and unlikely to be explained by genetic effects of the magnitude reported elsewhere.

## Conclusion

This research demonstrated a strong association between non-clinical maternal distress and child mental health problems between ages 3 and 11 years, which persisted after accounting for complex temporal effects of child mental health on subsequent maternal distress and baseline and time-varying confounding. Further research should investigate the influence of additional social determinants of mental health, such as family discord, a lack of support or social isolation (World Health Organization and Calouste Gulbenkian Foundation, 2014; Gariépy *et al.*, 2016). Proximal factors that might potentially mediate the observed negative effects of prolonged maternal distress on child mental health should also be explored, including insecure attachment, modelling maternal negative behaviours, maladaptive coping strategies for dealing with stress, reduced positive reinforcement, inconsistent discipline (Martins and Gaffan, 2000; Rishel, 2012) or adverse parenting practices (Elgar *et al.*, 2007; Turney, 2011*b*; Hutchings *et al.*, 2012; Tester-Jones *et al.*, 2017). Policymakers have acknowledged the importance of mental health problems in childhood, and in the UK this has been reflected in major investment in health services to treat and promote child and adolescent mental health (Department of Health, 2015), with a policy emphasis on the importance of early prevention (Murphy and Fonagy, 2013). Our results indicate that exposure to maternal psychological distress carries the potential to adversely affect child mental health. Furthermore, while services may focus on identifying and treating mothers with serious mental health problems, our findings show adverse effects at lower levels of distress, which are more common in the population. There are several complementary implications for the development of health and other services. First, family functioning should be considered by those offering primary care and adult mental health services to women who are mothers. This might include identifying whether families would benefit from parenting support and if children might also require services to reduce their risk of mental health problems (e.g. access to child care or treatment). Second, parenting support programmes should take into account the potential influence of maternal distress on children's mental health problems, and so also consider how common levels of maternal distress might be alleviated. Third, investment decisions relating to services which aim to support families through, for example, offering social support, skills training or advice on entitlement to state benefits, should take into account how reducing maternal distress might positively impact not only the mother's health but also potentially the mental health of her children.

## Supplementary material

For supplementary material accompanying this paper visit https://doi.org/10.1017/S0033291718001368. click here to view supplementary material
