# Supplementary material for: Temporal effects of maternal psychological distress on child mental health problems at ages 3, 5, 7 and 11: analysis from the UK Millennium Cohort Study
Source: Psychol Med. 2018 Jun 11;49(4):664–74. doi: 10.1017/S0033291718001368 (PMC6378410; doi:10.1017/S0033291718001368)
Supplement: Supplementary file 1 [file S0033291718001368sup001.docx]

**Temporal effects of maternal psychological distress on child mental health problems at ages 3, 5, 7 and 11: analysis from the UK Millennium Cohort Study**

**SUPPLEMENTARY APPENDIX:**

**S1: Latent Class Analysis**

Latent class analysis (LCA) is a data reduction technique designed to capture unobservable subgroups (or classes) using an array of observed characteristics. Individuals within the same class will be similar to each other, while individuals between classes will differ. Data on time-varying confounders representing family circumstances (household poverty, lone parent family, new sibling in the household, and maternal employment status) were summarised using a series of LCAs including time-varying confounders recorded from 3 years through to 11 years. The LCA for 3 years included only those recorded at that sweep, the LCA at 5 years included time-varying confounders at 5 years, the LCA at 7 years confounders at 7 years and, finally, the LCA at 11 years included time-varying confounders at 11 years. Missing items were automatically imputed during the LCA procedure under a missing at random assumption.

Models were run for both two and three latent classes, with cohort families assigned to the class that they had the highest probability of belonging to (Lanza, 2014). Selection of the optimal number of classes was based on both model fit and interpretability of the classes generated. Model fit comprised: class posterior probabilities (likelihood of members of an assigned class belonging to that class), item response probabilities (the probabilities that individuals displaying each of the time-varying confounders belonged to that latent class), entropy (the precision of membership assignment across all individuals). Each model was also assessed using Akaike information criterion (AIC) and Bayesian information criterion (BIC), where a lower value indicates greater parsimony.

Table S1.1 shows a summary of model fit for two and three class latent class solutions. Both two and three class models could fit the data well, although probabilities and entropy were more consistently high for the two class models, and a third class (labelled indeterminate) generated in three class models had lower probabilities than those shown for classes labelled adverse and non-adverse circumstances. BIC and AIC indices were larger for the two class solutions.

Tables S1.2 and S1.3 show, respectively for two and three class solutions, the item response probabilities at 3 years, 5 years, 7 years and 11 years. The two class solutions differentiated families with greater experiences of adversity. For three class solutions, although it was possible to identify classes with greater probabilities of adversity and no adversity, the third class represented a less interpretable indeterminate group, with characteristics that changed between sweeps.

In summary, the two class solution was both a good fit and interpretable, reflecting a dichotomy between those children who had experienced adverse circumstances versus those who had not.

**Table S1.1: Latent class analysis of time-varying confounders representing family circumstances: model fit for two and three class solutions**

|  | **3 years** | **5 years** | **7 years** | **11 years** |
| --- | --- | --- | --- | --- |
|  | (n=15381) | (n=15041) | (n=13681) | (n=13112) |
| **2 classes** |  |  |  |  |
| ***Posterior probabilities~*** |  |  |  |  |
| Class 1 (Not adverse circumstances) | 0.997 | 0.998 | 0.999 | 0.928 |
| Class 2 (Adverse circumstances) | 0.919 | 0.924 | 0.949 | 0.888 |
| ***Entropy**** | 0.89 | 0.89 | 0.93 | 0.71 |
| ***BIC*^** | 1322 | 817 | 804 | 170 |
| ***AIC^*** | 1281 | 777 | 765 | 131 |
| **3 classes** |  |  |  |  |
| ***Posterior probabilities~*** |  |  |  |  |
| Class 1 (Not adverse circumstances) | 0.929 | 0.881 | 0.982 | 0.949 |
| Class 2 (Adverse circumstances) | 0.937 | 0.896 | 0.915 | 0.858 |
| Class 3 (Indeterminate) | 0.735 | 0.728 | 0.758 | 0.589 |
| ***Entropy**** | 0.68 | 0.62 | 0.70 | 0.81 |
| ***BIC*^** | 210 | 250 | 242 | 104 |
| ***AIC^*** | 148 | 188 | 182 | 44 |

*~*1 indicates perfect assignment within that class; *1 indicates perfect assignment of all individuals to all classes; *^*lower values indicate a more parsimonious model

**Table S1.2: Latent class analysis of time-varying confounders representing family circumstances: probability estimates of two-class measures**

|  | **3 year** | | **5 year** | | **7 year** | | **11 year** | |
| --- | --- | --- | --- | --- | --- | --- | --- | --- |
|  | *Not Adverse* | *Adverse* | *Not Adverse* | *Adverse* | *Not Adverse* | *Adverse* | *Not Adverse* | *Adverse* |
|  |  |  |  |  |  |  |  |  |
| *New child in family* | 0.259 | 0.281 | 0.186 | 0.238 | 0.114 | 0.157 | 0.059 | 0.288 |
| *Lone parent family* | 0.065 | 0.431 | 0.082 | 0.454 | 0.111 | 0.46 | 0.191 | 0.356 |
| *Maternal unemployment* | 0.377 | 0.847 | 0.305 | 0.791 | 0.256 | 0.746 | 0.169 | 0.845 |
| *Low income household* | 0.038 | 0.999 | 0.037 | 0.999 | 0.021 | 0.999 | 0.024 | 0.721 |

**Table S1.3: Latent class analysis of time-varying confounders representing family circumstances: probability estimates of three-class measures**

|  | **3 year** | | | **5 year** | | | **7 year** | | | **11 year** | | |
| --- | --- | --- | --- | --- | --- | --- | --- | --- | --- | --- | --- | --- |
|  | *Not Adverse* | *Indeter-minate* | *Adverse* | *Not Adverse* | *Indeter-minate* | *Adverse* | *Not Adverse* | *Indeter-minate* | *Adverse* | *Not Adverse* | *Indeter-minate* | *Adverse* |
|  |  |  |  |  |  |  |  |  |  |  |  |  |
| *New child in family* | 0.149 | 0.490 | 0.187 | 0.347 | 0.02 | 0.247 | 0.242 | 0.000 | 0.167 | 0.052 | 0.902 | 0.228 |
| *Lone parent family* | 0.085 | 0.003 | 0.667 | 0.007 | 0.147 | 0.455 | 0.059 | 0.156 | 0.465 | 0.197 | 0.003 | 0.419 |
| *Maternal unemployment* | 0.104 | 0.925 | 0.856 | 0.515 | 0.07 | 0.816 | 0.535 | 0.002 | 0.785 | 0.192 | 0.781 | 0.857 |
| *Low income household* | 0.064 | 0.330 | 0.962 | 0.017 | 0.075 | 0.963 | 0.001 | 0.064 | 0.987 | 0.028 | 0.441 | 0.853 |

**S2: Marginal Structural Models** **(MSMs)**

When modelling longitudinal observational data, a common problem is how to account for confounding. Confounding is usually handled by including both the exposure and the confounders in standard regression models. However, standard regression models can be biased because they cannot appropriately adjust for confounding variables that change over time and may also be affected by prior exposure (See Figure 1). One approach developed to properly account for this time-dependent confounding is inverse probability of treatment weighting of marginal structural models (IPTW-MSM) (Robins *et al.*, 2000), in short MSM hereon.

In our analyses, we used inverse-probability-of-treatment weighting (IPTW) to control for confounding. The probability, conditional on past covariate history, of each subject’s having the observed level of maternal distress is predicted, and then each subject is weighted with the inverse of this probability in the outcome regression. The weights are computed using a logistic regression, as the exposure variable in this study is a binary variable (Hernan *et al.*, 2002). The IPTW weights are computed for each sweep and the overall weight is computed by taking the product of these sweep-specific weights. Although inverse weighting can be unstable and inefficient if there are extreme weights, this problem can be partially mitigated by using stabilised weights which were trimmed at the 5^th^ and 95^th^ centiles to remove the excessive influence of extreme values. For estimating the effects of exposure on the time-specific outcome we performed a stratified MSM. For example, when estimating the effect of maternal distress at 3, 5, 7 and 11 on child mental health problems at 11, we perform a MSM model that takes into account all the information from ages 3 to 11 of the child. The marginal structural model for the expected counterfactual outcomes, conditional on baseline exposure, is then fitted by regressing the observed outcome on the exposure at each time period but with each subject weighted by the stabilised IPTW:

$$SW\left( t \right)=\prod_{t=0}^{T} \left( \frac{f(X(t)|\bar{X}\left( t-1 \right))}{f(X(t)|\bar{X}\left( t-1 \right),\bar{L}\left( t \right),C)} \right)$$

Where *X* is the exposure, *t* is time or data collection sweep, *L* is time-varying confounder, *C* is time-invariant confounders and $\bar{X}, \bar{L}$ are the cumulative exposure up to time *t*, and the cumulative time-varying confounder effect up to time *t*. Robust variance estimation is used for standard errors to account for sampling error in the estimation of the weights.

MSMs allow inferences to be made about the effects of exposures on an outcome, properly accounting for time-varying confounding. The key assumption is that the baseline and time-varying covariates are sufficient to control for confounding at each point in time that exposure occurs.

The models take the form:

$$E(Y_{\left\{ x_{1},x_{2},x_{3},x_{4} \right\}})=\alpha_{0}+\beta_{1}x_{1}+\beta_{2}x_{2}+\beta_{3} x_{3}+\beta_{4} x_{4}$$

Where *Y* is child mental health problems (outcome) and $x$ is maternal distress (exposure). In the example shown, $Y_{\left\{ x_{1}x_{2}x_{3}x_{4} \right\}}$ is child mental health problems at 11 years for an individual that would have resulted under the hypothetical joint effects of maternal distress at ages 3, 5, 7 and 11 years to levels $x_{1}, x_{2},x_{3},x_{4}$, respectively. The variable $Y_{\left\{ x_{1}x_{2}x_{3}x_{4} \right\}}$ is sometimes referred to as a counterfactual outcome, as it is the outcome that would have resulted had the exposure, maternal distress at ages 3, 5, 7 and 11 years been set, possibly contrary to fact, to $x_{1}, x_{2},x_{3},x_{4}$. In the model, the effects on child mental health of joint exposure to maternal distress at ages 3, 5, 7 and 11 years are $\beta_{1},\beta_{2},\beta_{3},\beta_{4}$ respectively. The MSM is for the counterfactual outcome, not the observed outcome, and the expectation is marginalised over the entire population (the effect is marginal, not conditional on covariates). These risks estimated from MSM have causal interpretation (VanderWeele, 2015).

**S3: Unmeasured confounding sensitivity analysis**

A counterfactual-based, causal interpretation of estimates relies on the assumption that there is no unmeasured confounding between exposure and outcome, since unmeasured confounding may bias the estimation of true causal effects. An approach often referred to as “sensitivity analysis” or “bias analysis” over a range of sensitivity parameters allows causal inference to be made in the absence of observing all potential confounders. We used a method that specifies two measures of relative risk (*RR_XU_*, *RR_UY_*), where *RR_UX_* is the relative risk between the exposure and the unmeasured confounder and *RR_UY_* is the relative risk between the unmeasured confounder and the outcome (Ding and VanderWeele, 2016). This relative risk pair measures the strength of confounding between the exposure and outcome induced by the unobserved confounder (U). In the presence of unmeasured confounding, the true relative risk (*RR^True^*) must be at least as large as:

$$RR_{XY}^{True}\geq\frac{RR_{XY}^{Obs}}{\frac{RR_{UX}\times RR_{UY}}{RR_{UX}+RR_{UY}-1}}$$

Using this approach, we identified values of *RR_UX_* and *RR_UY_*, which would explain away the observed effects of maternal distress on child mental health. In Table S3.1, Column A shows the observed risks associated with maternal distress (exposure) on child mental health problems (outcome). Column B shows the joint bounding factor, or the level of unmeasured confounding required for a relative risk of unity between exposure and outcome. The column includes the two values, *RR_UX_* and *RR_UY_*, which are exchangeable. Both values demonstrate that the size of potential unmeasured confounding required to explain the exposure-outcome risks observed is large, given the range of socio-demographic factors accounted for in our analyses.

**Table S3.1: Unmeasured confounding sensitivity analysis**

|  | **Observed risks ratios**  $\boldsymbol{R}\boldsymbol{R}_{\boldsymbol{XY}}^{\boldsymbol{Obs}}$  **(A)** | **Joint bounding factor**  ***RR_UX_ and RR_UY_***  **(B)** |
| --- | --- | --- |
| **Child mental health problems at 3 years** | |  |
| Maternal distress at 3 years | 2.54 (2.30-2.81) | 3, 10 |
| **Child mental health problems at 5 years** | |  |
| Maternal distress at 3 years | 1.93 (1.64-2.28) | 2.5, 5 |
| Maternal distress at 5 years | 2.53 (2.16-2.96) | 3, 10 |
| **Child mental health problems at 7 years** | |  |
| Maternal distress at 3 years | 1.46 (1.21-1.76) | 1.5, 10 |
| Maternal distress at 5 years | 1.45 (1.23-1.71) | 1.5, 10 |
| Maternal distress at 7 years | 2.30 (1.97-2.67) | 4, 4 |
| **Child mental health problems at 11 years** | |  |
| Maternal distress at 3 years | 1.27 (1.08-1.49) | 1.3, 10 |
| Maternal distress at 5 years | 1.19 (1.01-1.41) | 1.3, 3 |
| Maternal distress at 7 years | 1.39 (1.20-1.62) | 1.5, 6 |
| Maternal distress at 11 years | 2.15 (1.89-2.45) | 3, 5 |

Interpretation of the sensitivity analysis can be illustrated with an example drawn from the table. For the association between maternal distress at 3 years on child mental health problems at 11 years, even if there was an unmeasured confounder that had a weak association with maternal distress at 3 years (RR=1.3), this confounder would need to be strongly associated with child mental health problems at 11 years (RR=10) for there to truly be no association ($RR_{XY}^{True}$=1) rather than the observed association ($RR_{XY}^{Obs}$=1.27). Although it may be possible to identify an unmeasured confounder associated with X (or Y) at RR=1.3, it is difficult to conceive of an unmeasured confounder that would have an association with Y (or X) as strong as 10.

**REFERENCES**

**Ding, P. & VanderWeele, T. J.** (2016). Sensitivity Analysis Without Assumptions. *Epidemiology* **27**, 368-377.

**Hernan, M. A., Brumback, B. A. & Robins, J. M.** (2002). Estimating the causal effect of zidovudine on CD4 count with a marginal structural model for repeated measures. *Statistics in Medicine* **21**, 1689-709.

**Lanza, S. D., J.; Huang ,L.; Wagner, A.; Collins, L.** (2014). LCA Stata Plugin Users' Guide: Version 1.1. Penn State: University Park: The Methodology Center.

**Robins, J. M., Hernan, M. A. & Brumback, B.** (2000). Marginal structural models and causal inference in epidemiology. *Epidemiology* **11**, 550-60.

**VanderWeele, T. J.** (2015). *Explanation in Causal Inference: Methods for Mediation and Interaction*. Oxford University Press.
